# Identification of disease-specific bio-markers through network-based analysis of gene co-expression: A case study on Alzheimer's disease

**DOI:** 10.1016/j.heliyon.2024.e27070

**Published:** 2024-03-01

**Authors:** Hexiang Zheng, Changgui Gu, Huijie Yang

**Affiliations:** Department of Systems Science, Business School, University of Shanghai for Science and Technology, Shanghai, 200093, China

**Keywords:** Gene co-expression network, Machine learning, Alzheimer's disease subtypes

## Abstract

Finding biomarker genes for complex diseases attracts persistent attention due to its application in clinics. In this paper, we propose a network-based method to obtain a set of biomarker genes. The key idea is to construct a gene co-expression network among sensitive genes and cluster the genes into different modules. For each module, we can identify its representative, i.e., the gene with the largest connectivity and the smallest average shortest path length to other genes within the module. We believe these representative genes could serve as a new set of potential biomarkers for diseases. As a typical example, we investigated Alzheimer's disease, obtaining a total of 16 potential representative genes, three of which belong to the non-transcriptome. A total of 11 out of these genes are found in literature from different perspectives and methods. The incipient groups were classified into two different subtypes using machine learning algorithms. We subjected the two subtypes to Gene Ontology analysis and Kyoto Encyclopedia of Genes and Genomes analysis with healthy groups and moderate groups, respectively. The two sub-type groups were involved in two different biological processes, demonstrating the validity of this approach. This method is disease-specific and independent; hence, it can be extended to classify other kinds of complex diseases.

## 1 Introduction

A large and increasing number of people are suffering from Alzheimer's disease (AD). In 2022, for instance, the number of AD patients worldwide reached 416 million [1], approximately six percent of whom are over 65 years old. The disease caused 1.9 million deaths. In fact, it is the main cause of sixty to seventy percent of dementia cases [[2], [3], [4]]. Detailed investigations show that it is closely related to many genes overlapping with those involved in alcohol intake, smoking, and dietary structure [5,6]. A poor lifestyle may increase the risk of Alzheimer's disease, but all daily factors take effect through genes. Hence, great efforts have been devoted to finding biomarker genes, identifying and classifying the disease, and shedding light on the occurrence mechanism [[7], [8], [9]].

Previous research on biomarkers based on gene co-expression network testing has mainly focused on Genome-Wide Association Studies (GWAS) and genomic information from single cells. Using GWAS, a total of 40 genes in patients are identified with mutations compared to healthy volunteers [[10], [11], [12], [13], [14], [15], [16], [17], [18], [19]]. These genes are believed to be closely related to AD. For instance, the gene APOE is attributed as the main disease-inducing gene in late-onset AD [20,21]. Kunkle et al. [19] searched for mutation sites in the whole genome using the GWAS meta-method, associated mutation sites with phenotypes through statistical methods such as T-test and GLM, and finally obtained five new biomarkers: the genes IQCK, ACE, ADAM10, ADAMTS1, and WWOX. Murdock et al. emphasized cell type-specific molecular perturbations in AD [22]. However, this method does not take into account the cumulative effect of some low-expression genes. In a cell, under environmental stimuli, a gene is expressed, the product of which may trigger the expression of several other genes. This kind of dynamic process connects the genes into a regulatory network [23], whose structure may amplify the cascades of low expressions to a big difference. Cao et al. [24] and Radhika et al. [25] have achieved remarkable success by applying Markov chains to genetic regulatory networks, enabling the identification of complex interactions between genes and proteins from a microscopic perspective. Hence, regulatory networks attract persistent attention in understanding the AD disease.

However, experimental identification of a large number of regulatory relationships is expensive and even impossible. Alternatively, researchers attempt to discover the functional relationships between genes embedded in co-expression records. The resulting network is called a Weighted Gene Co-Expression Network [26]. One currently used scheme involves calculating the cross-correlations between genes to represent their functional relationships, finding the optimal number of modules through a hierarchical clustering tree, and finally linking the genes in the modules with phenotypes through biological analysis to obtain biomarkers. For instance, Zakeri et al. [27] constructed co-expression networks for healthy people, patients with mild cognitive impairment, and AD patients, and separated them into a total of 5, 6, and 6 modules, respectively. The changes in the modules are identified and analyzed through functional enrichment analysis, suggesting a total of 9 biomarkers such as MBOAT1. Analogous analysis was conducted on peripheral blood samples, identifying a total of 7 biomarkers for AD. In this approach, the information in modular structure is adopted, but the detailed structural behaviors are lost in the clustering algorithm.

Many works demonstrate that the structural details of the regulatory network store rich information on complicated diseases. The genes in a regulatory network are usually clustered into modules participating in different biological functions, respectively [28,29]. Hub genes, i.e., those with a significant number of neighbors, usually play basic and crucial roles in normal biological processes in a cell [[28], [29], [30]] and have vital impacts on the development of diseases.

This study introduces an innovative strategy for biomarker discovery by leveraging structural properties of the regulatory network beyond modular behavior. Using gene expression records, we calculate cross-correlations between gene pairs, ranking them to identify a critical point that separates the curve into two branches. The resulting network is divided into modules, and within each module, the gene with the largest degree and the smallest average shortest path length is proposed as the representative biomarker. Applying this strategy to analyze gene expression profiles in Alzheimer's disease (AD) patients reveals that most proposed biomarkers overlap with those reported in the literature, with 11 out of 16 biomarkers identified in different papers using varied methods. Specifically, these 11 biomarkers are FGF1, S100A1, DAD1, RPS4Y1, PJA2, TCIRG1, PTPN14, PAK1, KLKB1, OR1G1, and PITRM1, respectively. Utilizing these biomarkers as input features, the Self-Organizing Map algorithm (SOM) efficiently classifies patients into subtypes, showing notable differences in gene expression, particularly in the incipient group. Furthermore, significant variations are observed in biological process analysis when applied to the moderate group.

## 2 Materials and methods

### 2.1 Gene co-expression network

The high-throughput gene co-expressions for healthy candidates and AD patients shared in the literature are used (data source from NCBI accession GSE1297 [31]). The volunteers include a total of 9 healthy people taken as reference. The patients are clinically separated into the incipient, moderate, and severe groups, containing 7, 8, and 7 samples, respectively. There are 22,284 RNA expression data per sample in the raw database, due to the existence of multiple RNAs corresponding to one DNA. We adopted the mean value strategy to merge multiple RNA data (corresponding to the same DNA). For each sample, the expression levels for a total of 13,235 genes are recorded, as shown in Table 1, a typical sheet of the records.

**Table 1:** Gene expression data and sample characteristics. Here, we provide the DNA expression data for each sample obtained after the transformation. This sample was taken using a gene chip platform identified as GPL96, which contains the correspondence between RNA probes and DNA. For the data of multiple RNAs corresponding to the same DNA, we adopt the strategy of averaging. Here, there is a total of 31 samples (on the middle side of the table), including nine healthy groups, seven incipient groups, eight moderate groups, and seven severe groups. For each sample, 13,235 gene expression data (on the left side of the table) are included. The different stages of the disease in each of the above samples are used for validation and are considered as true labels for the samples (on the top side of the table); the DNA expression data are used to calculate the differences in expression of the same genes in different samples.

Sample Group	Severe AD	Incipient AD	Incipient AD	…	Control	Control	Incipient AD
Sample characteristics - ntf	21	65.8	12	…	0.3	1.3	8
Sample characteristics - braak	6	5	6	…	1	2	5
Sample characteristics - mmse	4	25	29	…	28	27	21
Sample Label	GSM21203	GSM21204	GSM21205	…	GSM21231	GSM21232	GSM21233
PRPF8	1636.3	1725.2	1095.7	…	1141.7	1309.1	1330.5
CAPNS1	1694.4	2214.6	3375.2	…	3984.8	3075.6	2529.8
RPL35	3558.5	3929.4	4592.4	…	7090	4894.7	4129.9
EIF3D	1105.2	1156.6	934.7	…	1262.3	1271.3	1031.7
…	…	…	…	…	…	…	…
SCLY	397.25	506.05	622.05	…	301.53	259.1	348.1
ABCE1	397.25	506.05	622.05	…	525.3	495.85	640.95
SSBP2	504.5	668.1	522.9	…	321	492.35	444.8

Generally, the closely related genes that display significant differences cover only a small portion of all the genes. The other genes are independent of AD and accordingly fluctuate randomly. If we use all the expression levels to measure the differences between the subtypes, the true information will be merged with a large amount of noise. Hence, one should first filter out the genes not or weakly related to AD. There are several ways to search for disease-sensitive genomes. Herein, we adopt the simple variance and the information index classification measure (Appendix A) [32].

**Fig. 1:**
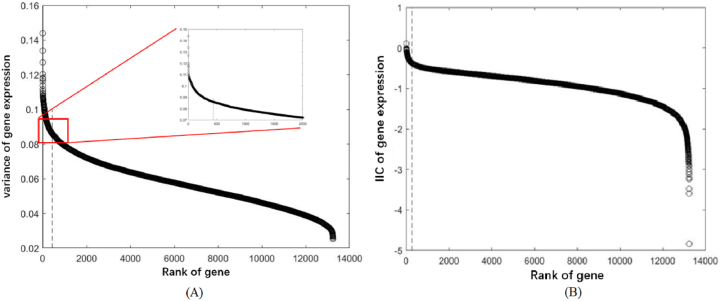
The sensitivities of genes to AD disease are displayed in descending order. (A) The variance: a critical point exists where the variance is 0.86, corresponding to gene number 438. A total of 438 genes are selected as sensitive genes. (B) The information index classification measure: a critical point also exists, but the number of genes screened out is too small, so this measure is not used.

Several methods are designed to screen out the sensitive genes. Herein, we use the descending order method. If we present the measures for the genes in descending order, the curve is generally separated by one or a few critical points into two or several parts that follow significantly different laws. It is reasonable to believe that the sensitive genes exhibit a behavior that is different from that of the weakly or even unrelated genes. Hence, the genes whose variances are larger than the first critical point are chosen to be sensitive genes (see Fig. 1 in the next section).

Let us denote the co-expression records of the sensitive genes for a specific group of volunteers g with,

(1) $$\begin{array}{c} R^{g}=\left\{R^{g}\left(m,n\right),m=1,2,\cdots ,m^{g};n=1,2,\cdots ,N\right\}\text{,} \end{array}$$

where g=h,i,m and s represent the healthy, incipient, moderate, and severe groups, respectively, $m^{g}$ the sample number of the corresponding group, N the total number of the sensitive genes. The cross-correlation matrices read,

(2) $$\begin{array}{c} C^{g}\left(n_{1},n_{2}\right)=\frac{\frac{1}{n}\left|\left|\sum_{m=1}^{m^{g}}\left(l_{n_{1}}^{m}-\mu_{n_{1}}\right)\left(l_{n_{2}}^{m}-\mu_{n_{2}}\right)\right|\right|}{\sqrt{\sum_{m=1}^{m^{g}}{\left(l_{n_{1}}^{m}-\mu_{n_{1}}\right)}^{2}\sum_{m=1}^{m^{g}}{\left(l_{n_{2}}^{m}-\mu_{n_{2}}\right)}^{2}}}\text{,} \end{array}$$

where $l_{n_{1}}^{m}\left(l_{n_{2}}^{m}\right)$ represents the $n_{1}\left(n_{2}\right)-th$ gene expression in the m−th sample, $\mu_{n_{1}}\left(\mu_{n_{2}}\right)$ the mean value of the $n_{1}\left(n_{2}\right)-th$ gene expression in all samples.

### 2.2 Bio-markers for the AD disease

The sensitive genes generally cluster into groups. Genes within a group are densely connected by strong linkages, forming a tight community. The groups are generally connected by sparse and weak linkages. Each group mainly participates in one or a few biological functions. The construction method implies that the genes within a group share a similar expression behavior, which is significantly different from that of the other groups. Accordingly, from each group, we obtained a specific gene as its representative. All the representatives are defined as the bio-markers of the AD disease.

Technically, the groups can be obtained using machine learning methods such as the K-means algorithm and the Louvain algorithm. In the K-means algorithm, one randomly selects a certain number of genes to serve as group centers, calculates the distances from each of the other genes to all the centers, and assigns the gene to the closest group. By calculating the distance within each group, new group centers are located and identified. This procedure is repeated until the distance between the centers of the groups saturates to a constant. In particular, we use the gap statistics in the K-means algorithm (Appendix B).

In the Louvain algorithm, every gene (node) is initially treated as an independent group. The modularity gain function is defined as,

(3) $$\begin{array}{c} S=\frac{1}{2N}\sum_{i,j}\left[C_{i,j}-\frac{k_{i}k_{j}}{2N}\right]\mu \left(i,j\right)\text{,} \end{array}$$

where S represents the gain of module, $k_{i}$ the sum of the weights of all edges connected to the i−th gene. μ(i,j) returns 1 if the i−th and j−th genes are in the same group, otherwise 0. At the beginning, we put the 1−th gene in other gene groups and calculate the modularity gains. The 1−th gene is added to the group with the largest modularity gain. The procedure is repeated until all the modules changes no longer.

Once a network is separated into groups, we cut the linkages between the groups to obtain the isolated groups. Within each group, for each gene, we calculate its degree (i.e., the summation of all the weights of the linkages) and the average of all the shortest path lengths from it to all the other genes. The representative of the group is defined to be the one that has the largest degree and the smallest average shortest path length.

### 2.3 Subtypes of the AD disease

Now each sample is described with a vector of expression levels for the representative genes, and we know its clinical subtype. The samples are then classified by means of the K-means [33], Self-Organizing Map [34], Competitive Neural Network [35], and Hierarchical Clustering algorithms [36]. In the prediction procedure, the healthy samples are not included; i.e., the algorithms are conducted only on the remaining 22 samples suffering from the AD disease.

The performance is evaluated through the Accuracy, Precision and Recall,

$$PRE=\frac{TP}{TP+FP}$$

(4) $$\begin{array}{c} REC=\frac{TP}{TP+FN} \end{array}$$

$$ACC=\frac{TP+TN}{TP+TN+FP+FN}\text{,}$$

from which one can define a balanced measure,

(5) $$\begin{array}{c} F1=2∙\frac{R_{precision}∙R_{recall}}{R_{precision}+R_{recall}}\text{,} \end{array}$$

where TN(FN) is the number of samples that are correctly (wrongly) not assigned to a specific subtype, and TP(FP) the number of samples that are correctly (wrongly) assigned to a specific subtype. The total number of samples equals to TP+TN+FP+FN. It should be noted that, for the sake of result stability, we conducted the experiment 50 times and subsequently performed statistical analysis on the outcomes.

## 3 Results

The values for the two measures of variance and IIC are presented in Fig. 1(A) and (B) in descending order, respectively. In the variance curve, a critical point appears, separating the curve into two branches obeying different laws. The genes whose variances are larger than this critical point are chosen as sensitive genes, with a total count of 438. As for the IIC measure, we also observe a transition in behavior, but we cannot obtain enough gene nodes to construct a network. Calculating gene expression variance in gene expression data and removing genes with low variance is a strategy to filter out those showing minimal differences between samples. This process enhances data richness, reduces noise, and focuses attention on genes with significant expression variations, often associated with key biological processes or disease states. The dimensionality reduction achieved by discarding low-variance genes contributes to more efficient analyses of large datasets.

**Fig. 2:**
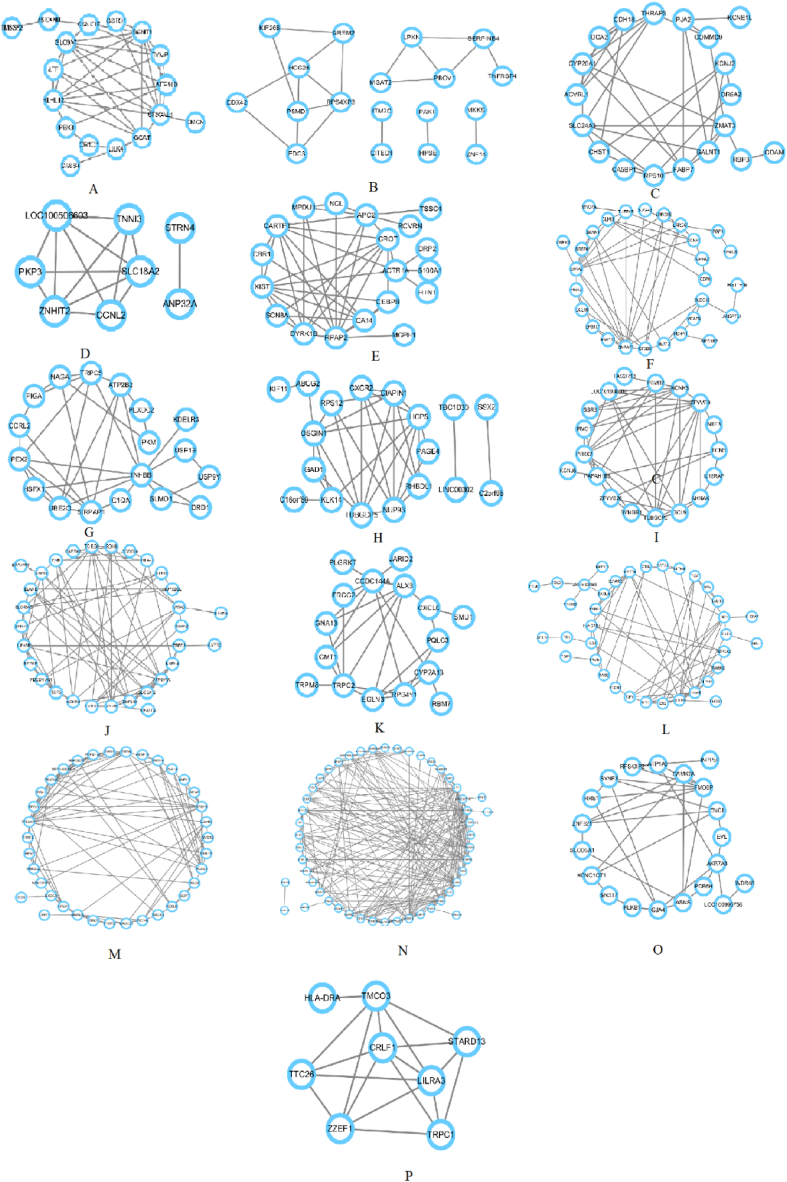
The constructed network for sub-graphs. We constructed subgraphs for each of the 16 modules. For instance, as shown in the small diagram (subfigure P) at the bottom of the figure, this gene group consists of HCG4, THRAP3, LCP2, TRDN, EMCN, TUBGCP5, LY6G6E, TAS2R13. The more important a single gene is in a module, the more contiguous it is with other genes in the graph. In this example, we can see that HCG4 has the most connected edges with other genes, so we consider HCG4 as the representative gene in this module. In vignette 2, this module is divided into two large groups and three small groups, and we consider that the large group plays a dominant role and therefore focus on analyzing the network properties of each gene node within the large group. In subfigure N, this module consists of 66 genes, and this relatively large module may function by the co-expression of multiple genes.

The constructed network is shown in Fig. 2(A–P), which is separated into a total of 16 modules by means of the K-means method. Some modules in the diagram are relatively large, and some are relatively small, while multiple groupings may occur within a single module. However, we uniformly capture only one gene in a module as a representative gene. In this method, the selection of a single representative gene from each module, based on maximum connectivity and shortest path length, demonstrates a degree of robustness and applicability. Genes with maximum connectivity imply more direct connections within the module, facilitating the capture of tight relationships and enhancing the representative gene's ability to reflect key features of the entire module. Choosing genes with the shortest path length ensures more direct and efficient relationships within the module, accurately capturing crucial information about gene interactions without being overly influenced by complex paths. The act of selecting a single representative gene simplifies the module's representation, reducing computational complexity while preserving essential information and presenting the overall nature of the module more clearly. Representative genes are designed to mirror the typicality of the entire module, offering better generalization regarding the complexity of interactions among genes within the module. The modules obtained with the Louvain method are neglected because the network is divided into many small pieces.

The corresponding 16 gene representatives are listed in Table 2, among which a total of 11 genes, including FGF1, S100A1, DAD1, RPS4Y1, PJA2, TCIRG1, PTPN14, PAK1, KLKB1, OR1G1, and PITRM1, were shown also to be closely related to Alzheimer's disease or neurological diseases by other research. Specifically, an investigation on the gene expression profile obtained with RNA sequencing (RNA-Seq) shows that when the expression of PJA2 decreases, some bio-markers will be expressed, such as APP, MAPT, GSAPAD, which is believed to inhibit axonal outgrowth and cell proliferation [37]. A blood sample experiment finds that the gene TCIRG1 is up-regulated by 20 percent in blood samples of AD patients at different stages; however, we have no idea about the underlying mechanism at the present time. Wilson et al. [38] suggest that the gene PTPN14 forms a complex with Kibra, the latter of which has been shown to be involved in the development of Alzheimer and memory performance [39]. Ma et al. [40] believe that the gene PAK1 affects cognition dysregulation, which can lead to Alzheimer's disease. Protein measurements for cognition in Alzheimer's disease [41] provide the clue for the significant relation of KLKB1 with the caudate nucleus (FDG CN). Olfactory disorders are believed to be ubiquitous in neurodegenerative diseases [42]. It is found that the gene OR1G1 affects the sense of smell by expressing the olfactory receptor protein 1G1. And the loss of PITRM1 function will induce Alzheimer [43]. A detailed analysis by Gélinas et al. [44] shows that the gene DAD1 and other genes are expressed in cells with amyloid-beta protein. Cohen et al. [45] show the evidence that the gene RPS4Y1 links with the Y-Linked Regulators and is over-expressed in sample cells. Afanador et al. [46] confirmed experimentally the pathological contribution of S100A1 to the biological process of AD. Shen et al. [47] report the significant up-regulation of CBR1 in the hippocampus of Alzheimer's mice. Tao et al. [48] through the GWAS method reveal the association of FGF1 with Alzheimer's disease.

**Table 2:** Community representative genes summary. Among the 16 community representative genes, only 13 are mapped to the protein interaction networks, and the remaining 3 belong to the non-transcriptome. Eleven of the 13 genes can be found in the literature to be associated with Alzheimer's disease.

Gene name	Description
FGF1	FGF1 is related to Alzheimer's disease and affects the survival of brain neurons
S100A1	S100A1 is involved in many cellular processes related to AD
DAD1	Related to amyloid-b protein and DAD1 expression when amyloid-b protein is stacked
RPS4Y1	Overexpression in cells of Alzheimer
PJA2	Critical for PKA-mediated long-term memory process
TCIRG1	It is directly involved in T cell activation and is related to Alzheimer's disease
PTPN14	Mediates the dephosphorylation of -catenin at the adhesion junction
PAK1	Can directly phosphorylate BAD and protect cells from apoptosis
KLKB1	Plays a role in the renin-angiotensin system by converting prorenin to renin, and the increase in adrenaline leads to the production of amyloid in Alzheimers disease
OR1G1	Olfactory receptor 1G1, decreased sense of smell may be a precursor to Alzheimers disease
PITRM1	When it accumulates in the mitochondria, it can degrade amyloid A4 (APP) protein, indicating that it is related to Alzheimer's disease

By means of four clustering algorithms, we classify the samples, the performances of which are shown in Table 3. Among the four clustering algorithms, the SOM algorithm performs better and is more suitable for this task. A tentative explanation is that it properly mimics the neuronal response to stimulation in the brain. It has the greatest positive response to the directly stimulated neuron. As the distance increases, the response to stimulation will gradually weaken until it becomes a suppressive one. While gene expression can promote the expression of related genes on the one hand, on the other hand, it can inhibit the expression of genes with a lower degree of correlation. In the network, the expressions of genes that have a greater degree of association with the hub gene have greater changes, while those for the genes at the edge of the network have smaller changes.

**Table 3:** Evaluation table of the effect of each clustering algorithm. The categories in the above table represent the number of target categories or the number of activated neurons, and the group is the actual label of the sample. For example, for the K-means algorithm, we set the number of classification targets to 3 and 5, respectively. When the number of classification targets is 3, the evaluation indexes calculated for each group are shown above; when the number of classification targets is 5, we consider that if the proportion of the incipient group is the largest in the group, the label of this group is considered to be incipient, so we divide the five obtained groups into three eventually. And for the SOM algorithm, we use a combination of 2*3 neurons.

Methods	Categories	Group	Precision	Recall	F1 value	ACC
K-means	Three	incipient	60%	43%	0.45	59%
		moderate	63%	63%	0.63	
		severe	56%	71%	0.67	
	Five	incipient	67%	57%	0.45	59%
		moderate	56%	63%	0.63	
		severe	57%	57%	0.67	
Competitive Neural Network	Three	incipient	63%	71%	0.69	59%
		moderate	62%	63%	0.63	
		severe	50%	43%	0.44	
Self-Organizing Map	2 * 3	incipient	63%	71%	0.69	68%
		moderate	63%	63%	0.63	
		severe	83%	71%	0.73	
Hierarchical Clustering	Five	incipient	50%	57%	0.55	64%
		moderate	83%	63%	0.66	
		severe	63%	71%	0.69	

**Fig. 3:**
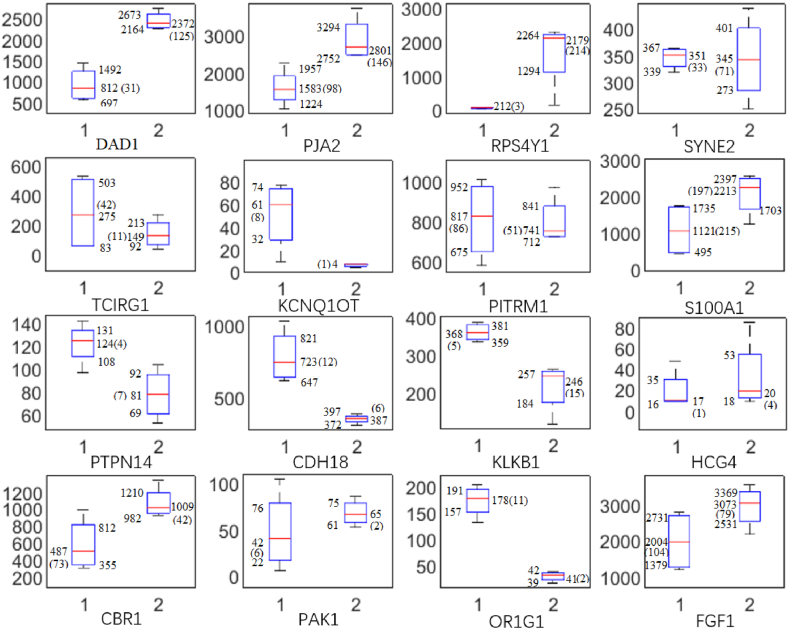
Gene expression difference table. The above table is divided into 16 sub-graphs. Taking DAD1 as an example, the bottom 1 of the sub-graph represents incipient type-1, and the bottom 2 represents incipient group type-2. The histogram on the left of the sub-graph represents the expression data of four samples, with the expression level of type 2 being 2.5 times that of type 1. For OR1G1, the expression level of type 1 is 5 times that of type 2. There are genes with similar expression levels, such as SYNE2. Although the expression levels fluctuate greatly, the overall mean values are similar. The numbers in the figure represent the upper quartile, mean (variance), and lower quartile, respectively. For example, in DAD1, the four numbers are 1492, 812 (31), and 697, respectively.

The SOM algorithm separates the incipient patients into two types, named type-1 and type-2. The expressions of 14 of the 16 representative genes are significantly different (see Fig. 3), i.e., a total of 7 genes are significantly up-regulated, while the other 7 genes are significantly down-regulated.

Gene Ontology analysis (GO analysis) also displays a clear difference in biological processes and cellular components between the type-1 and type-2 samples, as listed in Table 4. The type-1 (type-2) samples are mainly involved in the negative (active) regulation of biological processes and cellular processes. The type-1 mainly constitutes the extracellular area, such as the cell periphery, cell plasma membrane, and cell surface, while the type-2 mainly constitutes the intracellular area, such as intracellular organelles.

**Table 4:** GO analysis and KEGG analysis difference table (partial). A clear difference between type-1 and type-2 is shown in the above table (part). Type-1 and type-2 show no difference from the control group (healthy group). These two incipient types are significantly different from the moderate group. Specifically, no differences were revealed for the two subtypes of the incipient group when analyzed with the control group; however, when analyzed with the moderate group, it was observed that the entries of the incipient group type-1 differed from the control group mainly in cellular composition, specifically in the plasma membrane and cell surface, while the incipient group type-2 differed from the control group mainly in the intracellular area.

Analysis	Incipient type − 1 and Control	Incipient type − 2 and Control
Biological Process	GO:0065007 biological regulation biological process	GO:0065007 biological regulation biological process
	GO:0048522 positive regulation of cellular process	GO:0048522 positive regulation of cellular process
Molecular Function	GO:0005515 protein binding	GO:0005515 protein binding
Cellular Function	GO:0044459 plasma membrane part	GO:0005622 intracellular cellular component
	GO:0044456 synapse part	GO:0044446 intracellular organelle part
Analysis	Incipient type − 1 and Moderate	Incipient type − 2 and Moderate
Biological Process	GO:0048523 Negative regulation of cellular process	Go:0048522 positive regulation of cellular process
	GO:0048519 Negative regulation of biological process	GO:0048518 Positive regulation of biological process
Molecular Function	GO:0043168 anion binding	GO:0043168 anion binding
Cellular Component	GO:0071944 cell periphery	GO:0005622 intracellular cellular component
	GO:0009986 cell surface	GO:0044424 intracellular part
KEGG analysis	hsa04145: Phagosome	hsa04110: Cell cycle
		hsa04020: Calcium signaling pathway
		hsa04151: PI3K-Akt signaling pathway
		hsa04971: Gastric acid secretion

Kyoto Encyclopedia of Genes and Genomes analysis (KEGG analysis) indicates that type-1 is mainly associated with phagocytic pathways, which, however, are not related to Alzheimer's disease. For type-2, the cell cycle, calcium signaling, and PI3K-Akt signaling pathways have been documented to be related to AD. Comparing the two subtypes with the incipient group and healthy groups shows that the two subtypes and the healthy group are basically similar in biological processes. Hence, it is not possible to distinguish between type-1 and type-2 in terms of biological progress.

## 4 Conclusion

In this study, we present a novel approach for classifying samples afflicted by Alzheimer's disease, offering a multi-faceted contribution to the field.

Initially, we introduce a network-based screening method designed to identify potential biomarkers from gene expression data. This approach involves constructing a cross-correlation network to intricately model expression relationships among sensitive genes, capturing the complexities of gene interactions. The network is then partitioned into distinct modules, revealing the organizational structure of gene expression. Within each module, representative genes are extracted based on maximum connectivity and the smallest average shortest path length, providing a simplified yet powerful approach for identifying potential biomarkers.

Our integrated approach of network analysis and modularization paves the way for biomarker research, emphasizing the importance of extracting crucial information from complex gene expression data. Furthermore, we propose a set of 16 representative genes as biomarkers for different disease states. Remarkably, 11 out of the proposed 16 biomarkers align with findings in various papers using different methodologies and perspectives.

Employing machine learning with 16 gene representatives, particularly the Self-Organizing Map (SOM) algorithm, enhances classification, effectively distinguishing diverse disease states. Notably, the incipient group, combining two subtypes, poses challenges in detection when mixed with healthy samples. Disease progression indicates a convergence to a common moderate state, revealing two more refined stages within the incipient group. This offers insights for targeted treatment and a deeper understanding of disease development. Compared to existing research on subtype detection using artificial intelligence [[49], [50], [51], [52]], our method excels in achieving stable predictive results with small sample sizes. Additionally, utilizing gene expression data directly from the samples enhances the direct representation of sample states.

The use of gene co-expression networks in biomarker extraction provides advantages by comprehensively understanding global gene interactions, partitioning networks for finer identification of function-specific gene sets, and optimizing biomarker discovery. This approach exhibits noise resistance, ensuring stability even in the presence of experimental errors. In contrast, machine learning offers significant advantages for Alzheimer's disease subtype classification, handling high-dimensional gene expression data to comprehend disease complexity, with strong generalization capabilities extending learned patterns to new samples. This enables efficient processing of large-scale data for rapid and accurate patient classification, supporting precision medicine research and making it a crucial tool for precise comprehension and intervention in Alzheimer's disease.

Our work introduces a novel network-based screening method for identifying biomarkers in Alzheimer's disease. This multi-faceted contribution involves intricate gene expression network analysis, proposing a set of representative genes, and leveraging machine learning for precise disease classification. The approach provides valuable insights into disease progression and offers a streamlined strategy for biomarker identification, showcasing potential applications beyond Alzheimer's disease. Obviously, detailed studies on a large number of samples and other types of complex diseases are required to confirm the high performance of our method.

## Data availability

Data will be made available on request.

## Ethics declarations

Review and/or approval by an ethics committee was not needed for this study because this study only used publicly available sources.

## Funding

This work is supported by the 10.13039/501100001809National Natural Science Foundation of China (Grant Nos. 12275179 and 11875042), and 10.13039/100007219Natural Science Foundation of Shanghai (Grant No. 21ZR1443900).

## CRediT authorship contribution statement

**Hexiang Zheng:** Writing – original draft, Software, Methodology, Formal analysis. **Changgui Gu:** Writing – review & editing, Funding acquisition, Formal analysis. **Huijie Yang:** Writing – review & editing, Formal analysis.

## Declaration of competing interest

The authors declare that they have no known competing financial interests or personal relationships that could have appeared to influence the work reported in this paper.

## Appendix A

The information index classification measure:

$$IIC\left(i\right)=\frac{1}{2}\frac{\left|\mu_{1}\left(i\right)-\mu_{2}\left(i\right)\right|}{\sigma_{1}\left(i\right)+\sigma_{2}\left(i\right)}+\frac{1}{2}\ln\frac{{\sigma_{1}\left(i\right)}^{2}+{\sigma_{2}\left(i\right)}^{2}}{2\sigma_{1}\left(i\right)\sigma_{2}\left(i\right)}\text{,}$$

where $\mu^{c}\left(i\right)$ and $\sigma^{c}\left(i\right)$ are the mean and the variance of the i−th gene in the reference (c=2) and the disease (c=1) samples.

## Appendix B

The gap statistics in the K-means algorithm,

$$D_{k}=\sum_{x_{i}\epsilon C_{k}}\sum_{x_{j}\epsilon C_{k}}{\left|\left|x_{i}-x_{j}\right|\right|}^{2}$$

$$W_{k}=\sum_{k=1}^{K}\frac{1}{2n_{k}}D_{k}$$

$${Gap}_{n}\left(k\right)=E_{n}^{*}\left(\log\;W_{k}\right)-\log\;W_{k}\text{,}$$

where $D_{k}$ is the sum of distances within the sub-network, k the optimal number of sub-network, $x_{i}\left(x_{j}\right)$ the vector composed of the expression quantity of i(j)−th gene in all samples in group $C_{k}$, $W_{k}$ the variance quantity, $n_{k}$ a total number of all genes, $E_{n}^{*}$ the expectation of $\log\;W_{k}$. An optimal separation is reached when ${Gap}_{n}\left(k\right)$ saturates to a maximum constant. In general, the elbow method is commonly used, but since this method requires manual selection of curve inflection points, which in turn adds an artificial factor. The GAP statistic, on the other hand, needs to be calculated to get the maximum K, without adding the human factor. Therefore, we choose the GAP statistic as the criterion for the number of classifications in the K-means algorithm.
